# Anti-inflammatory properties of a novel peptide interleukin 1 receptor antagonist

**DOI:** 10.1186/1742-2094-11-27

**Published:** 2014-02-03

**Authors:** Boris Klementiev, Shizhong Li, Irina Korshunova, Oksana Dmytriyeva, Stanislava Pankratova, Peter S Walmod, Laura K Kjær, Mattias S Dahllöf, Morten Lundh, Dan P Christensen, Thomas Mandrup-Poulsen, Elisabeth Bock, Vladimir Berezin

**Affiliations:** 1 Laboratory of Neural Plasticity, Department of Neuroscience and Pharmacology, University of Copenhagen, Blegdamsvej 3, DK-2200 Copenhagen, Denmark; 2 Section of Endocrinological Research, Department of Biomedical Sciences, University of Copenhagen, Blegdamsvej 3, DK-2200 Copenhagen, Denmark; 3 Department of Molecular Medicine and Surgery, Karolinska Institute, Solna (L1:00), SE-171 77 Stockholm, Sweden

**Keywords:** Amyloid-β, Antagonistic peptide, Experimental autoimmune encephalitis, Inflammation, Interleukin 1, LPS, Pancreatic islets

## Abstract

**Background:**

Interleukin 1 (IL-1) is implicated in neuroinflammation, an essential component of neurodegeneration. We evaluated the potential anti-inflammatory effect of a novel peptide antagonist of IL-1 signaling, Ilantide.

**Methods:**

We investigated the binding of Ilantide to IL-1 receptor type I (IL-1RI) using surface plasmon resonance, the inhibition of Il-1β-induced activation of nuclear factor κB (NF-κB) in HEK-Blue cells that contained an IL-1β-sensitive reporter, the secretion of TNF-α in macrophages, protection against IL-1-induced apoptosis in neonatal pancreatic islets, and the penetration of Ilantide through the blood–brain barrier using competitive enzyme-linked immunosorbent assay (ELISA). We studied the effects of the peptide on social behavior and memory in rat models of lipopolysaccharide (LPS)- and amyloid-induced neuroinflammation, respectively, and its effect in a rat model of experimental autoimmune enchephalomyelitis.

**Results:**

Ilantide bound IL-1RI, inhibited the IL-1β-induced activation of NF-κB, and inhibited the secretion of TNF-α *in vitro*. Ilantide protected pancreatic islets from apoptosis *in vitro* and reduced inflammation in an animal model of arthritis. The peptide penetrated the blood–brain barrier. It reduced the deficits in social activity and memory in LPS- and amyloid-treated animals and delayed the development of experimental autoimmune enchephalomyelitis.

**Conclusions:**

These findings indicate that Ilantide is a novel and potent IL-1RI antagonist that is able to reduce inflammatory damage in the central nervous system and pancreatic islets.

## Introduction

Interleukin 1 (IL-1) is a key mediator of the acute-phase inflammatory response and has been implicated as the mediator of tissue dysfunction and destruction in chronic inflammatory diseases such as amyotrophic lateral sclerosis, diabetes and rheumatoid arthritis. IL-1 is also believed to mediate neuroinflammation in neurodegenerative conditions, including Alzheimer’s disease (AD) [1]. IL-1 signals via IL-1 receptor type I (IL-1RI), which binds both the IL-1α and IL-1β isoforms [2]. Once bound to the receptor, the IL-1 receptor accessory protein (IL-1RAcP) is recruited, allowing nuclear factor κB (NF-κB) and mitogen-activated protein kinase (MAPK) signaling to trigger the expression and release of a multitude of inflammatory mediators, including chemokines and cytokines. IL-1 antagonists encompass IL-1 receptor antagonist (IL-1Ra), IL-1-neutralizing antibodies and the IL-1 trap, a linear fusion protein between the soluble IL-1 receptor (sIL-RI) and IL-1RAcP. IL-1Ra binds to IL-1RI, but it does not initiate signaling, because it does not engage IL-1RAcP [3,4]. These biologics are indicated for the treatment of autoinflammatory diseases and rheumatoid arthritis and have shown promising results in clinical trials for other inflammatory conditions. Anakinra (Ana) is a recombinantly produced protein that contains the *N*-terminal, methionylated, nonglycosylated version of human IL-1Ra, which competitively blocks the actions of IL-1 without any detectable agonist activity. Ana has been used for the treatment of rheumatoid arthritis, adult-onset Still’s disease, systemic onset juvenile idiopathic arthritis, osteoarthritis and type 2 diabetes mellitus [5,6]. However, the relatively poor penetration of the blood–brain barrier (BBB) [7] restricts therapeutic use of the current macromolecular IL-1 antagonist proteins for the treatment of neuroinflammation [8].

In the present study, we identified and characterized a novel synthetic peptide, ten amino acids long, termed *Ilantide*, which is derived from the human IL-Ra *N*-terminal domain involved in interactions with IL-1RI. We hypothesized that Ilantide would compete with IL-1R signaling, thereby inhibiting inflammatory responses induced by various conditions *in vitro* and *in vivo*. Indeed, we found that Ilantide bound to IL-1RI and inhibited the IL-1-induced activation of NF-κB and secretion of tumor necrosis factor α (TNF-α) by macrophages. It protected pancreatic islets from IL-1β-induced apoptosis and reduced inflammation in collagen-induced arthritis (CIA). The peptide penetrated the BBB, ameliorated decline in social activity and memory in lipopolysaccharide (LPS)- and amyloid-treated animals and delayed the development of experimental autoimmune encephalomyelitis (EAE).

## Materials and methods

### Peptides and recombinant proteins

The Ilantide peptide (SGRKSSKMQA), scrambled peptide1 (KQSAGKRSMS), scrambled peptide2 (KASQKGMSRS) and Ilantide peptide with a reversed sequence (AQMKSSKRGS) were purchased from Schafer-N (Copenhagen, Denmark). The peptides were synthesized using the fluorenylmethyloxycarbonyl protection strategy on TentaGel resin (Rapp Polymere, Tübingen, Germany). The peptides were synthesized as either monomers (Ilantide-m) or dendrimers composed of four monomers coupled to a lysine backbone (Ilantide-t) and were further purified by gel filtration using Sephadex G-10 (Amersham Biosciences, Uppsala, Sweden). The peptides were at least 85% pure according to estimation by high-performance liquid chromatography. The recombinant proteins (that is, IL-1β, IL-6, TNF-α, interferon γ (IFN-γ), IL-1Ra and the ectodomain of IL-1RI) were purchased from R&D Systems (Minneapolis, MN, USA). Ana (Kineret) was obtained from Amgen (Thousand Oaks, CA, USA).

### Surface plasmon resonance analysis

The binding analysis was performed using a Biacore 2000 instrument (GE Healthcare Life Sciences) that contained 150 mM NaCl as running buffer at 25°C with 10 mM sodium phosphate (pH 7.4). The flow rate was 5 μl/min during immobilization. Ilantide peptide, IL-1β or IL-1Ra was immobilized on a CM4 sensor chip according to the manufacturer’s instructions, after which the recombinant ectodomain of IL-1RI (R&D Systems) was injected. The neural cell adhesion molecule (NCAM) immunoglobulin modules 1 and 2 (Ig1 and Ig2) were used as a negative control protein [9]. The data were analyzed using nonlinear curve-fitting with the manufacturer’s software.

### Cell cultures

HEK-Blue IL-1β cells that contained an IL-1β-sensitive reporter (InvivoGen, San Diego, CA, USA) and HEK-Blue IL-6 cells that contained an IL-6-sensitive reporter (InvivoGen) were grown at 37°C with 5% CO_2_ in Dulbecco’s modified Eagle’s medium (DMEM) supplemented with 5% (vol/vol) fetal calf serum (FCS), 4.5 g/L glucose, 2 mM GlutaMAX medium, 100 U/ml penicillin, 100 μg/ml streptomycin (all obtained from Gibco BRL/Life Technologies, Taastrup, Denmark), 100 μg/ml zeocin, 200 μg/ml hygromycin and 100 μg/ml Normocin (all purchased from InvivoGen).

A murine alveolar macrophage cell line, AMJ2-C8 (American Type Culture Collection, Borås, Sweden), was routinely grown at 37°C with 5% CO_2_ in DMEM supplemented with 5% (vol/vol) FCS, 0.5% (v/v) 2-[4-(2-hydroxyethyl)piperazin-1-yl]ethanesulfonic acid (HEPES) (Gibco BRL), 2 mM GlutaMAX, 100 U/ml penicillin and 100 μg/ml streptomycin.

Cerebellar granule neurons (CGNs) were prepared from 7-day-old Wistar rats (from Charles River Laboratories, Sulzfeld, Germany; or from Hygind Møllegård Erik Møllegaard Hansen, Ejby, Denmark) as previously described [10]. Briefly, the cerebella were cleared of meninges and blood vessels, roughly homogenized by chopping and trypsinized. The cells were washed in the presence of DNAse I and soybean trypsin inhibitor (Sigma-Aldrich, St Louis, MO, USA), and cellular debris was pelleted by centrifugation. The fibroblastoid mouse cell line L929 (LVN; European Collection of Cell Cultures, Porton Down, UK) was routinely grown at 37°C with 5% CO_2_ in DMEM supplemented with 10% (vol/vol) FCS, 2 mM GlutaMAX, 100 U/ml penicillin and 100 μg/ml streptomycin.

### Rat islet isolation and culture

Neonatal rat islets were isolated from 3- to 6-day-old outbred Wistar rats (Taconic, Ejby, Denmark) as previously described [11]. Islets were precultured and maintained in RPMI 1640 medium (Gibco/Life Technologies) supplemented with 20 mmol/L HEPES buffer, 0.038% (wt/vol) NaHCO_3_, 100 U/ml penicillin, 100 g/ml streptomycin and 10% (vol/vol) newborn calf serum (NCS) at 37°C in humidified atmospheric air. Prior to experimentation, the islets were randomly distributed to 24-well dishes and incubated for 2 hours in RPMI 1640 medium supplemented as described above, but with 0.5% (vol/vol) NCS relevant reagents were added as indicated.

### Nuclear factor κB activation

HEK-Blue IL-1β cells allow the monitoring of the activation of the NF-κB pathway specifically in response to IL-1β. They express a NF-κB-inducible secreted embryonic alkaline phosphatase (SEAP) reporter gene. The binding of IL-1β to its receptor IL-1RI on the surface of HEK-Blue IL-1β cells triggers a signaling cascade that leads to the activation NF-κB and the subsequent production of SEAP. HEK-Blue IL-1β cells were grown as described above. The cells were seeded onto a 96-well plate at a density 3.5 × 10^5^ cells/ml. For the NF-κB activation assay, the cells were treated with 1.2 pM IL-1β alone or together with sIL-1RI, IL-1Ra or various concentrations of Ilantide. After 24 hours of incubation at 37°C, 150 μl of the cell supernatants were added to each well of a 96-well plate together with 50 μl of QUANTI-Blue (InvivoGen), incubated at 37°C for 40 minutes and measured in an enzyme-linked immunosorbent assay (ELISA) reader at 600 nm to determine the expression levels of reporter genes activated by NF-κB.

### Signal transducer and activator of transcription 3 activation

HEK-Blue IL-6 cells were grown as described above. IL-6 cells allow the monitoring of the activation of the signal transducer and activator of transcription 3 (STAT3) pathway specifically in response to IL-6. They express a STAT3-inducible SEAP reporter gene. For the STAT3 activation assay, the cells were treated with 1.2 pM IL-6 alone or together with various concentrations of Ilantide. TNF-α (1.2 pM) was added as a control ligand for both the NF-κB and STAT3 assays. After 24-hour incubation at 37°C, 150 μl of HEK-Blue cell supernatant were added to each well of a 96-well plate together with 50 μl of QUANTI-Blue (InvivoGen), incubated at 37°C in the dark for 40 minutes and measured in an ELISA reader at 600 nm to determine the expression level of the secreted SEAP.

### Tumor necrosis factor α secretion

AMJ2-C8 macrophages were seeded into six-well multidishes at a density of 2.5 × 10^5^ cells/well, and sIL-1RI, IL-1Ra or various concentrations of Ilantide were added to the cultures. Treatment with 100 μM hydrocortisone (Sigma-Aldrich, Brøndby, Denmark) was used as a positive control. After 10-minute incubation at 37°C, 1.2 pM IL-1β or IFN-γ (R&D Systems) was added to activate the macrophages. L929 cells were seeded onto a 96-well plate (Nunc, Roskilde, Denmark) at a density 0.2 × 10^2^ cells/ml. Both cell cultures were incubated for 24 hours at 37°C. Conditioned medium from macrophages was collected and added to the L929 cell cultures together with actinomycin D (0.6 μg/well; Sigma-Aldrich). TNF-α is known to induce classic apoptosis in L929 cells in the presence of actinomycin D [12]. After 24-hour incubation at 37°C, 20 μl of 3-(4,5-dimethylthiazol-2-yl)-5-(3-carboxymethoxyphenyl)-2-(4-sulfophenyl)-2*H*-tetrazolium (Promega, Madison, WI, USA) were added to each well and incubated at 37°C in the dark for 45 minutes, then measured in an ELISA reader at 490 nm. To calculate the amount of TNF-α in the conditioned medium, fibroblasts were treated with standard concentrations of TNF-α (R&D Systems).

### Cell survival and apoptosis

#### Pancreatic islets and nitric oxide production

For cell death detection, rat islets were incubated with 150 pg/ml IL-1β (BD Pharmingen, San Diego, CA, USA) alone or in combination with various concentrations of Ana and Ilantide for 24 hours at 37°C in humidified atmospheric air. Rat islet cell death was assessed by cell death detection ELISA (Roche, Lund, Sweden) as previously described [13]. As a surrogate measure of nitric oxide production, Griess reagent (1:1 aqueous mixture of 0.1% naphthylethylenediamine hydrochloride and 1% sulfanilamide + 5% H_3_PO_4_) was mixed in a 1:1 ratio with 100 μl of supernatant samples to determine the nitrite content in supernatants from the cell death assays described above.

#### Primary neurons

Cerebellar granule neurons were plated at a density of 1 × 10^5^ cells/cm^2^ on poly-L-lysine-coated, eight-well Permanox Lab-Tek chamber slides (Nunc) in Neurobasal-A medium supplemented with 2% (vol/vol) B27 (Gibco BRL), 0.5% (vol/vol) GlutaMAX, 100 U/ml penicillin and 100 μg/ml streptomycin. The CGN survival assay was performed as previously described [14]. The neurite outgrowth assay was also performed as previously described [15].

### Animal studies

Male Wistar rats (Charles River Laboratories) weighing 150 to 200 g on the day of arrival in the laboratory were housed two per cage. Juvenile rats (3 weeks old, weight 40 to 50 g) were housed in groups of three. All of the animals were kept under standard conditions (23°C, 50% humidity, 12:12-hour light-dark cycle) with free access to food and water. All of the experiments were performed according to European Union legislation with licenses from the Danish Animal Experiments Inspectorate (2008/561-1539, 2008/561-1524 and 2009/561-1686). The number of animals utilized in the respective experimental groups was kept to a minimum, and all of the work was conducted in a manner designed to cause the least harm and suffering to the animals.

#### Pharmacokinetics

Biotinylated Ilantide-t (Schafer-N) was administered to adult male Wistar rats subcutaneously (s.c.) at a dose of 10 mg/kg. Twenty minutes, sixty minutes, one hundred forty minutes, five hours and thirty hours after peptide administration, blood samples (approximately 300 μl) were collected from the orbital plexus under fentanyl/droperidol/midazolam anesthesia (0.002%/0.14%/0.014% wt/vol) (Department of Experimental Medicine, University of Copenhagen, Denmark) in ethylenediaminetetraacetic acid–coated tubes (BD, Plymouth, UK), and 50 to 110 U/ml aprotinin (Calbiochem, San Diego, CA, USA) was added immediately to the collected blood. The samples were subsequently centrifuged at 1,500 × *g* for 15 minutes. Cerebrospinal fluid (CSF) was collected from the cisterna magna 30 minutes after peptide administration as described previously [16]. Plasma and CSF samples were stored at -80°C.

The concentrations of Ilantide-t in plasma and CSF samples were measured by performing a competitive ELISA on amino 96-well plates (Nunc). The bottoms of the wells were coated with biotinylated bovine serum albumin (Sigma-Aldrich) diluted in coating buffer (0.1 M Na-carbonate buffer, pH 9.6) to a concentration of 1 μg/ml, and 100 μl were applied to each well. The plates were incubated overnight at 4°C and then washed three times in washing buffer (PBS with 0.1% vol/vol Tween 20, pH 7.4). One volume of diluted sample or a standard with a known concentration of the biotin-labeled Ilantide was pipetted into the wells of a mixing Protein LoBind Eppendorf Plate (Eppendorf, Hamburg, Germany) and incubated with three volumes of streptavidin-peroxidase (Dako Denmark A/S, Glostrup, Denmark) diluted 1:10,000 in washing buffer. After preincubation for 30 minutes, 100 μl of the resulting incubation mixture were transferred onto the prepared ELISA plate and then incubated for 1 hour at 37°C. The plate was then washed three times in washing buffer, and 3,3′,5,5′-tetramethylbenzidine substrate (Kem-En-Tec Diagnostics, Taastrup, Denmark) was added (100 μl/well) for 5 to 10 minutes at room temperature. Color development was terminated by the addition of 2 M H_2_SO_4_ (100 μl/well), and absorbance was read at 450 nm on a Wallac Victor 1420 multilabel counter (PerkinElmer, Hvidovre, Denmark). Peptide concentrations in the samples were determined using a standard curve. Samples from four to six animals were used for each time point and run in duplicate. Two independent determinations were performed.

#### Collagen-induced arthritis in rats

CIA was induced by bovine collagen type II (CII; Sigma-Aldrich) solubilized in 0.05 M acetic acid (2 mg/ml), then emulsified 1:1 with complete Freund’s adjuvant (CFA) (Sigma-Aldrich) that contained 1.0 mg/ml heat-inactivated *Mycobacterium tuberculosis*. While the animals were under inhalation anesthesia (3% isoflurane; Baxter, Allerød, Denmark), 250 μl of the emulsion that contained 250 μg of CII and 125 μg of *M. tuberculosis* were injected intradermally at the tail base (day postimmunization 0 (dpi 0)). On dpi 8, before the onset of clinical signs, all of the animals were randomly divided into two groups, with 20 rats per group, and dosed daily for 8 days (dpi 8 to 15) with Ilantide s.c. (10 mg/kg) or vehicle s.c. (1.0 ml/kg PBS). Clinical evaluations were performed on dpi 7 to 16. An observer who was blinded to the treatment groups evaluated the severity of arthritis by employing the following grading system: 0 (no redness or swelling of the foot), 1 (slight redness in the foot or redness and swelling in single interphalangeal joints), 2 (moderate swelling and redness in the ankle and metatarsal part of the foot), 3 (marked swelling and redness of the entire foot, with restricted use of the foot during locomotion) and 4 (marked swelling and redness of the entire foot, with no use of the foot during locomotion). CIA typically involves only the hindlimbs, and the arthritic index was defined as the sum of the two limb scores. The animals were killed when the severity of arthritis reached a score of 7. The differences in the area under the clinical score curve during the period of observation between the control and peptide-treated groups were calculated, with baseline defined as the start of treatment (that is, 0).

#### Lipopolysaccharide-induced impairment of social behavior

The peripheral administration of LPS, the active fragment of bacterial endotoxin, results in an increase in the plasma levels of all proinflammatory cytokines and behavioral depression [8]. LPS-induced behavioral depression was assessed by measuring the reduction of the duration of social interaction during 4-minute sessions. Six- to seven-week-old rats were handled for five days, habituated to the test cages and the experiment room and trained for social interaction with three-week-old juvenile rats introduced into the test cage for four minutes two days before the actual experiment. On the day of the experiment, the rats were intraperitoneally injected (i.p.) with saline or LPS (*Escherichia coli* 0111:B4; Sigma-Aldrich) dissolved in saline (250 μg/kg). Immediately after the i.p. injection, Ilantide-t (Ila; 10 mg/kg, equivalent to 2.1 μmol/kg), Ana (100 mg/kg, equivalent to 5.8 μmol/kg) or saline was injected subcutaneously. The volume of all of the injections was 1.0 ml/kg. Six experimental groups were used: vehicle i.p./vehicle s.c. (veh/veh; *n* = 10), vehicle i.p./Ilantide-t s.c. (veh/Ila; *n* = 8), vehicle i.p./Ana s.c. (veh/Ana; *n* = 9), LPS i.p./vehicle s.c. (LPS/veh; *n* = 10), LPS i.p./Ilantide-t s.c. (LPS/Ila; *n* = 11) and LPS i.p./Ana s.c. (LPS/Ana; *n* = 11). The injections and weighing of the rats were performed immediately after the first test session (0 h), and the animals were tested and weighed again 2, 4, 6, 8, and 24 hours later. Different juvenile animals were presented on each occasion. Social interaction consisted of anogenital sniffing, licking and chewing the fur of the juveniles by the experimental animals and was monitored with a video camera. Animals that showed less than 40 seconds of investigation of the juveniles during the baseline session were excluded. The duration of social interaction at each time point is expressed as the percentage of baseline values and was averaged for each experimental group. Social interaction was scored by an observer who was blinded to the treatment of the animals. For evaluation of levels of pro- and anti-inflammatory cytokines in blood, rats were injected with LPS and then immediately with either vehicle (*n* = 8) or Ilantide-t (*n* = 8) as described above. In addition, a control group of animals (*n* = 4) was treated with Ilantide-t alone (control). Blood samples were collected at 0, 2 and 6 hours after injections as described for the pharmacokinetics study and stored at -20°C until use. The levels of IL-6, IFN-γ and IL-10 were determined using corresponding rat ELISA kits according to the manufacturer’s protocols (all from BD Biosciences, San Diego, CA, USA).

#### Intracerebroventricular administration of the Aβ_25–35_ peptide

Aggregates of Aβ_25–35_ (Bachem AG, Weil am Rhein, Germany) were prepared by incubating the peptides at a concentration of 3 μg/μl in sterile water for 4 days at 37°C. Subsequently, 5 μl of aggregated Aβ_25–35_ were injected intracerebroventricularly (1.2 μl/min) with a 10-μl Hamilton syringe using the following coordinates: 0.8 mm posterior to the bregma, 1.5 mm lateral to the sagittal suture and 3.8 mm beneath the surface of the brain. The procedure was performed while the animals were under anesthesia (i.p. fentanyl-fluanisone/midazolam at 0.3 ml/100-g animal; that is, 23.6 μg of fentanyl, 0.75 mg of fluanisone and 375 μg midazolam per 100-g animal).

#### Social recognition test

Social memory in animals reflects the ability of individuals to recognize conspecifics as familiar or unfamiliar. The social recognition test is a measure of short-term memory and is suitable for studies of AD-like dementia because it evaluates the function of neural structures that involve the cholinergic system, which is impaired in AD [17,18]. The animals were handled for 5 days, habituated to the test cage and trained to interact with a juvenile rat. Three groups of rats were used: a control group of untreated rats (*n* = 11), an amyloid-/vehicle-treated group (*n* = 13) and an amyloid/Ilantide-t-treated group (*n* = 12). Aggregated amyloid-β protein fragment 25 to 35 (Aβ_25–35_; 15 μg) was injected into the lateral ventricle. Ilantide-t (10 mg/kg) or vehicle (1 ml/kg saline) was injected subcutaneously on days 7, 9, 11, 13, 15, 17 and 19 after Aβ administration. The social recognition test was conducted on day 20. Every rat was tested three times. The first and second trials (T_1_ and T_2_) were performed with the same juvenile. In the third trial (T_3_), the rats were tested with a new juvenile. The trial lasted 4 minutes with a 30-minute intertrial interval. During each trial, the investigative behavior of the adult toward the juvenile (that is, licking, sniffing and chewing the fur of the juvenile) was monitored with a video camera and scored by an observer who was blinded to the treatment of the animals. Animals that showed less than 40 seconds of investigation of the juvenile during the initial trial were excluded. Social memory was estimated as a recognition ratio (RR). RR_familiar_ was calculated as T_2_/(T_1_ + T_2_). T_1_ and T_2_ are the time spent investigating the juvenile animal during the first and second trials. The time spent investigating a novel juvenile (T_3_) was measured to confirm that the effect seen with the familiar juvenile was specific to cognition. In this case, RR_new_ was calculated as T_3_/(T_1_ + T_3_). An RR value significantly less than the theoretical value of 0.5, calculated by a one-sample *t*-test, was taken as an indication of the presence of social memory.

#### Experimental autoimmune encephalomyelitis

Experimental autoimmune enchephalomyelitis was induced in female Lewis rats by guinea pig myelin basic protein (MBP) emulsified with CFA (Sigma-Aldrich) that contained 1.0 mg/ml heat-inactivated *M. tuberculosis*. While the animals were under inhalation anesthesia (3% isoflurane; Baxter), 200 μl of an emulsion that contained 200 μg of MBP were subcutaneously injected at the tail base (dpi 0). Between dpi 0 and 21, weight and clinical signs of EAE were recorded daily for all of the animals. Clinical signs were scored as follows: 0 (no abnormality), 0.5 (weak tail), 1 (limp tail), 2 (mild palsy of one or both hind legs), 3 (severe palsy of one or both hind legs), 4 (complete paralysis of one or both hind legs), 5 (paralysis of one or both hindlimbs and the beginning of paralysis of the forelimbs) and 6 (moribund). Animals with a clinical score ≥4 were immediately killed. From dpi 10 forward, the animals were treated once daily for 5 consecutive days with Ilantide-t (10 mg/kg, 1 ml/kg, s.c.) or PBS (1.0 ml/kg s.c.). Only animals that reached a clinical score ≥1 before dpi 14 were included in the study.

### Data analysis

The statistical analysis was performed using one-way analysis of variance (ANOVA) followed by the Newman-Keuls multiple-comparisons *post hoc* test or two-way ANOVA followed by the Bonferroni *post hoc* test, a nonparametric *t*-test and a one-sample *t*-test when appropriate (GraphPad Prism 5 software; GraphPad Software, La Jolla, CA, USA).

## Results

### Ilantide, a peptide modeled after the IL-1Ra binding site for IL-1RI, binds to the ectodomain of IL-1RI with an affinity comparable to the cytokine

**Figure 1:**
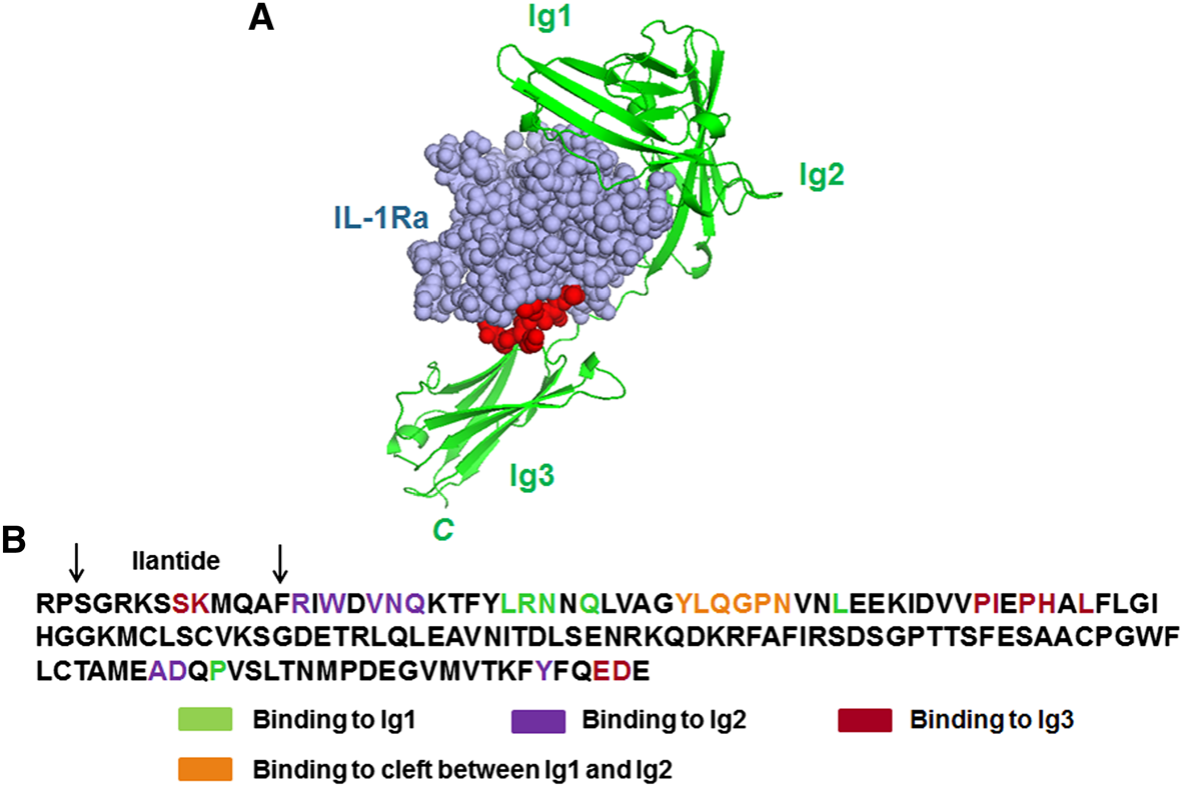
**Design of the peptide Ilantide. (A)** Tertiary structure model of the complex between interleukin 1 receptor antagonist (IL-1Ra) (space filled with purple) and IL-1 receptor type I (IL-1RI) (backbone and secondary structures in green). The location of the Ilantide peptide sequence is shown in red. **(B)** The IL-1Ra sequence with the mapped IL-1RI binding site and Ilantide motif is shown.

**Figure 2:**
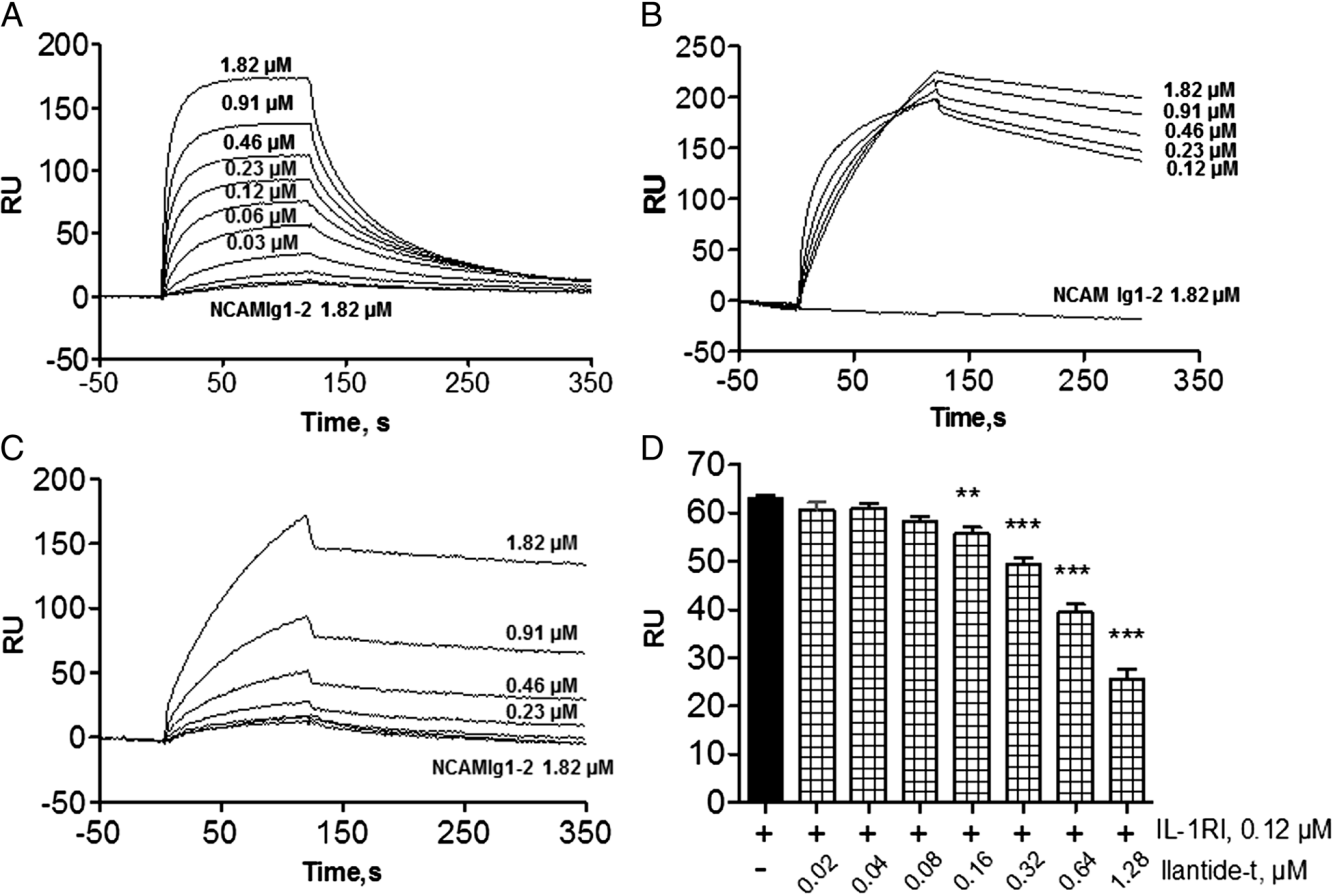
**Interaction between Ilantide, interleukin 1β, and interleukin 1 receptor antagonist and soluble IL-1 receptor type I.** Binding was studied using surface plasmon resonance analysis. Approximately 2,500 to 3,000 resonance units (RUs) of interleukin 1β (IL-1β) **(A)**, IL-1 receptor antagonist (IL-Ra) **(B)** and Ilantide-t **(C)** were immobilized on a sensor chip. Binding is expressed as the response difference between the binding of IL-1 receptor type I (IL-1RI) to the sensor chip with the immobilized peptide, IL-1β, or IL-Ra and a blank sensor chip. No binding of any of the tested proteins and Ilantide-t was observed to the double-immunoglobulin (Ig) module (Ig1-Ig2) of the neural cell adhesion molecule (NCAM) (negative control). **(D)** Binding of the IL-1RI ectodomain to immobilized IL-1β in the presence of the indicated concentrations of Ilantide-t. The results from three experiments are expressed as mean RU ± SEM. **P < 0.01 and ***P < 0.001 compared with binding of IL-1RI alone to IL-1β (one-way analysis of variance followed by the Newman-Keuls *post hoc* test).

Using a model of the tertiary structure of the IL-1Ra–IL-1RI complex [PDB:1IRA] [19], we designed a peptide ten amino acids long from the *N* terminus of IL-1Ra, termed *Ilantide* (*IL*-1 *an*tagonistic pep*tide*), a part of the IL-1Ra protein that interacts with the Ig3 module of the type I receptor (Figure 1). We studied the binding of the peptide to IL-1RI by surface plasmon resonance (SPR) analysis. IL-1β and IL-Ra were used as positive controls. Ilantide-t, IL-1β and IL-1Ra bound to IL-1RI (Figures 2A to 2C). The calculated affinity and rate constants of these interactions are shown in Table 1. IL-1Ra and Ilantide-t bound IL-1RI with apparent K_d_ values within the same order of magnitude (4.8 × 10^-8^ M and 2.75 × 10^-8^ M, respectively), whereas IL-1β had a lower affinity for the receptor (1.71 × 10^-7^ M). As a control, we also measured the binding of Ilantide-t, IL-1β and Il-1Ra to a control Ig-like module molecule that consisted of the double-Ig module of NCAM and found no significant binding (Figures 2A to 2C). Ilantide-t dose-dependently inhibited the binding between IL-1RI and IL-1β (Figure 2D), indicating that IL-1β and Ilantide competed for the same binding site in IL-1RI.

**Table 1:** **Affinity binding and rate constants of Ilantide, interleukin 1β and interleukin 1 receptor antagonist to interleukin 1 receptor type I**^**a**^

**Ligands**	**k**_**a **_**(M**^**-1 **^**s**^**-1**^)	**k**_**d **_**(s**^**-1**^)	**K**_**d **_**(M)**
Ilantide	1.84 ± 0.20 × 10^5^	2.69 ± 0.58 × 10^-3^	2.75 × 10^-8^ ± 7 .86 × 10^-9^
IL-1Ra	1.47 ± 5.53 × 10^5^	3.62 ± 0.65 × 10^-3^	4.80 × 10^-8^ ± 1.32 × 10^-8^
IL-1β	8.56 ± 2.79 × 10^5^	7.53 ± 2.03 × 10^-4^	1.71 × 10^-7^ ± 7.07 × 10^-9^

^**a**^IL-1β, interleukin 1β; IL-1Ra, interleukin 1 receptor antagonist.

### Ilantide inhibits the interleukin 1β-induced activation of nuclear factor κB

**Figure 3:**
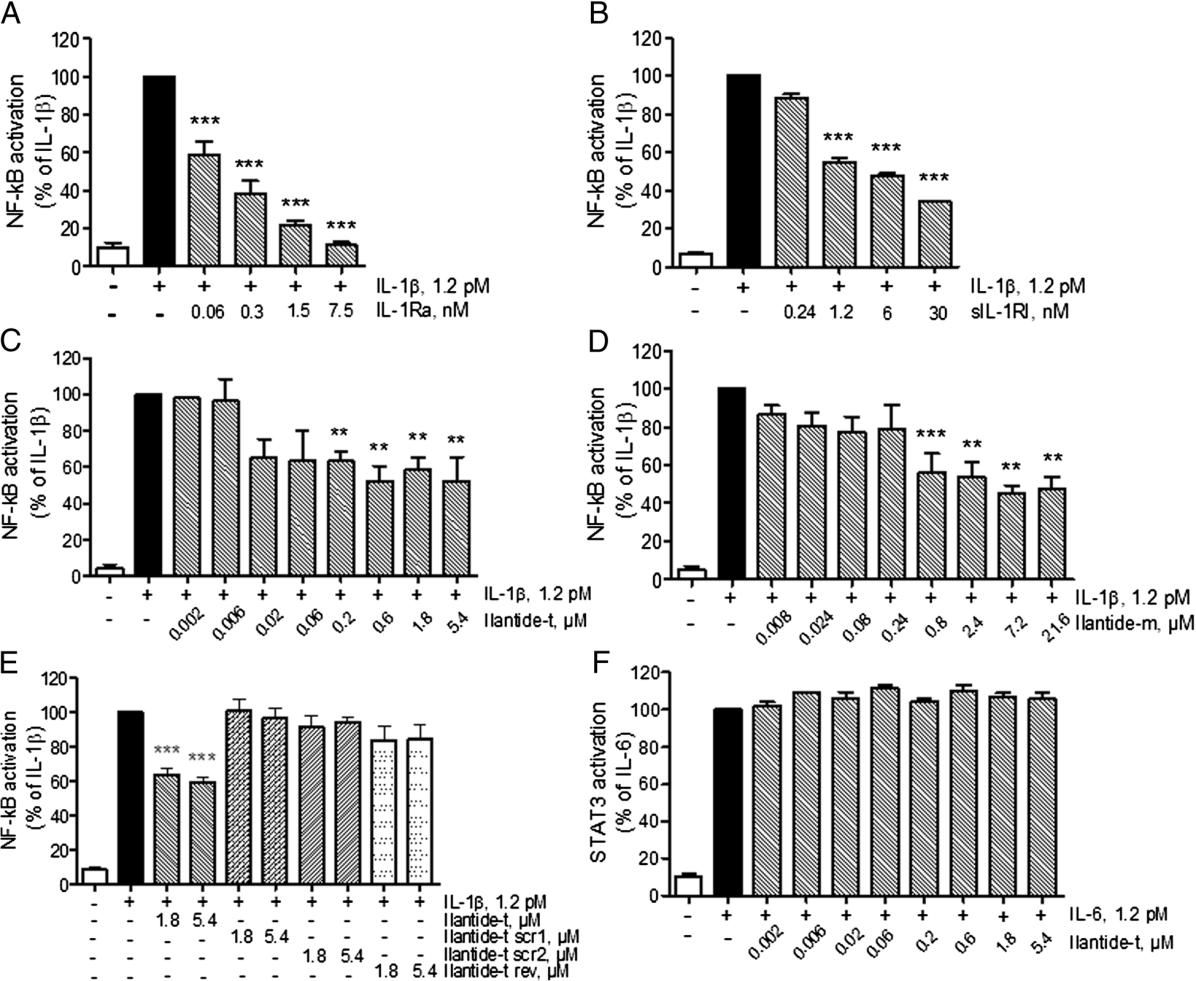
**Effect of Ilantide on nuclear factor κB activation induced by interleukin 1β.** HEK cells that overexpressed interleukin 1 receptor type I (IL-1RI) or IL-6 receptor (IL-R6) were first treated with the Ilantide peptides, control peptides, IL-1 receptor antagonist (IL-1Ra) or soluble IL-1RI (sIL-1RI), after which IL-1β-induced nuclear factor κB (NF-κB) or IL-6-induced signal transducer and activator of transcription 3 (STAT3) activation was determined by enzyme-linked immunosorbent assay through the induction of a reporter gene that expressed secreted embryonic alkaline phosphatase under the control of the interferon β minimal promoter fused to five NF-κB binding sites. **(A)** IL-1β-induced NF-κB activation was inhibited by IL-1Ra. **(B)** IL-1β-induced NF-κB activation was inhibited by sIL-1RI. **(C)** IL-1β-induced NF-κB activation was inhibited by Ilantide-t. **(D)** IL-1β-induced NF-κB activation was inhibited by Ilantide-m. **(E)** Control peptides with scrambled or reversed sequences did not inhibit IL-1β-induced NF-κB activation. **(F)** Ilantide-t did not inhibit IL-6-induced STAT3 activation. The results from four independent experiments are expressed as percentage ± SEM, in which control cells treated with either IL-1β or IL-6 alone were set at 100%. **P < 0.01 and ***P < 0.001 compared with IL-1β-treated or IL-6-treated controls (one-way analysis of variance followed by Newman-Keuls *post hoc* test).

The NF-κB signaling pathway plays a central role in the regulation of IL-1-mediated inflammation [20]. We assumed that Ilantide might inhibit NF-κB activation induced by IL-1β because of the ability of Ilantide to outcompete IL-1RI–IL-1β binding (Figure 2D). To test this hypothesis, HEK cells that overexpressed IL-1RI were used. NF-κB activation was induced by IL-1β. The cells were treated with Ilantide, control peptides, IL-1Ra or sIL-1RI, and the activation of NF-κB was determined by ELISA. Similarly to IL-1Ra and sIL-1RI, both Ilantide-m and Ilantide-t dose-dependently inhibited NF-κB activation induced by IL-1β (Figures 3A to 3D), whereas the scrambled and reversed Ilantide-t peptides had no effect (Figure 3E). In contrast, Ilantide-t did not inhibit STAT3 activation induced by IL-6 (Figure 3F), suggesting that Ilantide specifically inhibits NF-κB activation induced by IL-1β through binding to IL-1RI.

### Ilantide inhibits interleukin 1β-induced secretion of tumor necrosis factor α in macrophages

**Figure 4:**
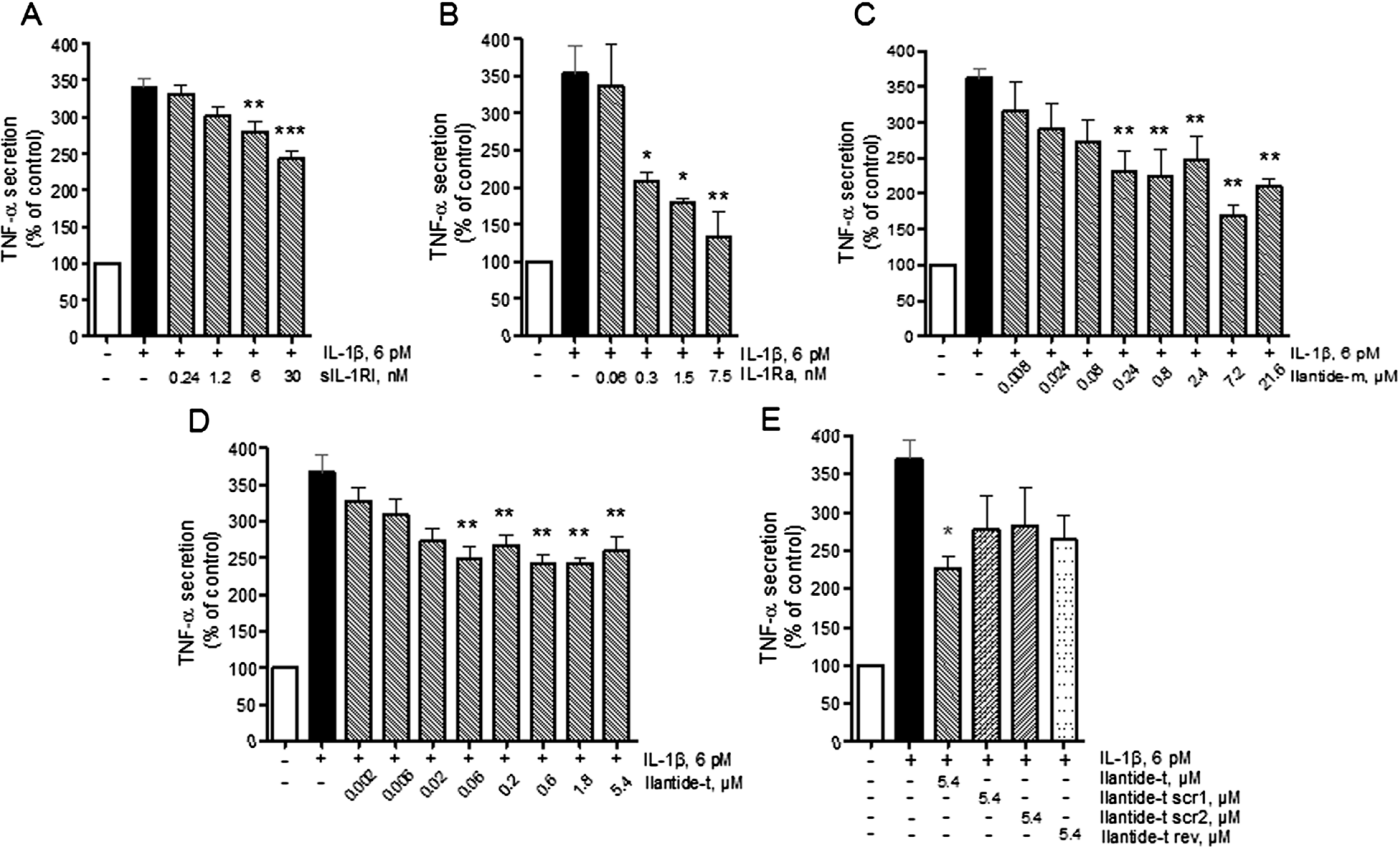
**Effect of Ilantide on tumor necrosis factor α release from interleukin 1β-activated macrophages.** AMJ2-C8 macrophages were exposed to interleukin 1β (IL-1β) and treated with Ilantide peptides, control peptides, IL-1 receptor antagonist (IL-1Ra) or soluble IL-1RI (sIL-1RI) for 24 hours. The conditioned medium from macrophages was used to treat fibroblast cells for 24 hours, and the amount of tumor necrosis factor α (TNF-α) in the conditioned medium was evaluated on the basis of fibroblast cell survival in the presence of actinomycin D. **(A)** TNF-α release from IL-1β-activated macrophages was inhibited by IL-1Ra. **(B)** TNF-α release from IL-1β-activated macrophages was inhibited by sIL-1RI. **(C)** TNF-α release from IL-1β-activated macrophages was inhibited by Ilantide-t. **(D)** TNF-α release from IL-1β-activated macrophages was inhibited by Ilantide-m. **(E)** Scrambled (scr) or reverse sequence control peptides did not inhibit TNF-α release from IL-1β-activated macrophages. The results from four independent experiments are expressed as percentage ± SEM with untreated controls set at 100%. *P < 0.05, **P < 0.01 and ***P < 0.001 compared with macrophages treated with IL-1β only (one-way analysis of variance followed by Newman-Keuls *post hoc* test).

Macrophages are activated by T lymphocytes, resulting in the production of proinflammatory cytokines (for example, TNF-α and IL-1β). These cytokines trigger inflammatory cascades, thus leading to the activation of inflammation [21,22]. To test whether Ilantide, IL-1β or IL-1Ra would reduce TNF-α release from IL-1β-activated macrophages, we used a mouse-derived macrophage cell line, AMJ2-C8. The cells were pretreated with Ilantide, control peptides, IL-1Ra, sIL-1R or hydrocortisone and stimulated by IL-1β or IFN-γ. The conditioned medium from the macrophages was used to treat L929 cells, which, in the presence of actinomycin D, are sensitive to picomolar concentrations of TNF-α [12]. The amount of TNF-α in the conditioned medium was calibrated against fibroblasts exposed to standard concentrations of TNF-α. As expected, IL-1β-induced macrophage TNF-α production was completely inhibited by 100 μM hydrocortisone (data not shown). Similarly to IL-1Ra and sIL-1RI, Ilantide-m and Ilantide-t significantly inhibited IL-1β-induced TNF-α secretion in macrophages (Figures 4A to 4D), whereas the scrambled Ilantide peptides had no statistically significant effect (Figure 4E).

### Ilantide promotes the survival of primary neurons

**Figure 5:**
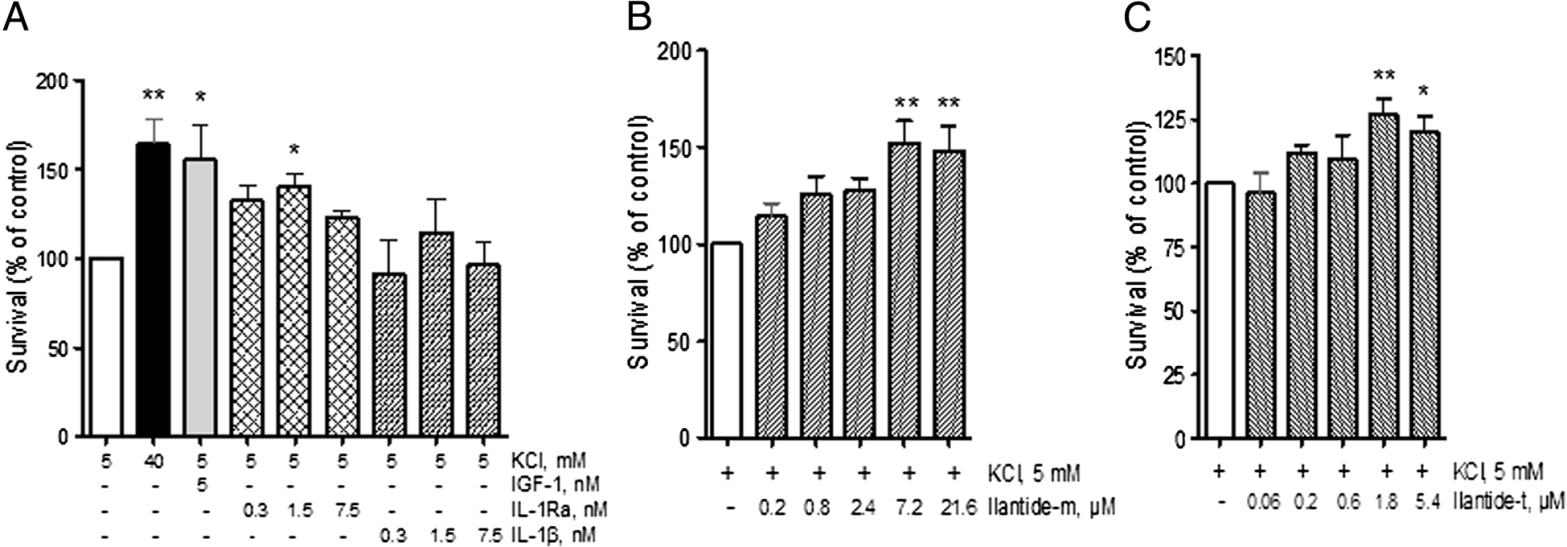
**Effect of Ilantide and interleukin 1 receptor antagonist on survival of cerebellar granule neurons induced to undergo apoptosis.** The neurons were allowed to differentiate for 7 days in a high-potassium (40 mM) medium before apoptosis was induced by changing to a low-potassium medium (5 mM). Survival was estimated 48 hours later. **(A)** The effect of insulin-like growth factor 1 (IGF-1), interleukin 1 receptor antagonist (IL-1Ra) and IL-1β on cerebellar granule neuron (CGN) apoptosis induced by 5 mM KCl. **(B)** The effect of Ilantide-m on CGN apoptosis induced by 5 mM KCl. **(C)** The effect of Ilantide-t on CGN apoptosis induced by 5 mM KCl. The results from at least four independent experiments are expressed as mean ± SEM with untreated cultures induced to undergo apoptosis by 5 mM KCl set at 100%. *P < 0.05 and **P < 0.01 compared with untreated cultures induced to undergo apoptosis (one-way analysis of variance followed by Newman-Keuls *post hoc* test).

IL-1 mediates neuronal cell death during acute brain injury [23], and IL-1Ra has neuroprotective effects on cerebral ischemia, excitotoxicity and brain trauma in various rodent models [24,25]. Therefore, we tested whether Ilantide or IL-1Ra promotes neuronal cell survival. Cerebellar granule neurons from 7-day-old rats were differentiated for 7 days in a high-potassium medium, after which the neurons were grown for 2 days in a low-potassium medium either the presence or absence of insulin-like growth factor 1 (IGF-1; positive control), Ilantide-m, Ilantide-t, IL-1Ra or IL-1β. Cell death induced by potassium withdrawal was inhibited by IGF-1 (Figure 5A). Treatment with IL-1Ra, Ilantide-m and Ilantide-t, but not IL-1β, concentration-dependently promoted CGN survival (Figure 5). These results indicate that the inhibition of IL-1RI in CGNs leads to an increase in neuronal cell survival.

### Ilantide stimulates neuritogenesis in cultures of primary neurons

**Figure 6:**
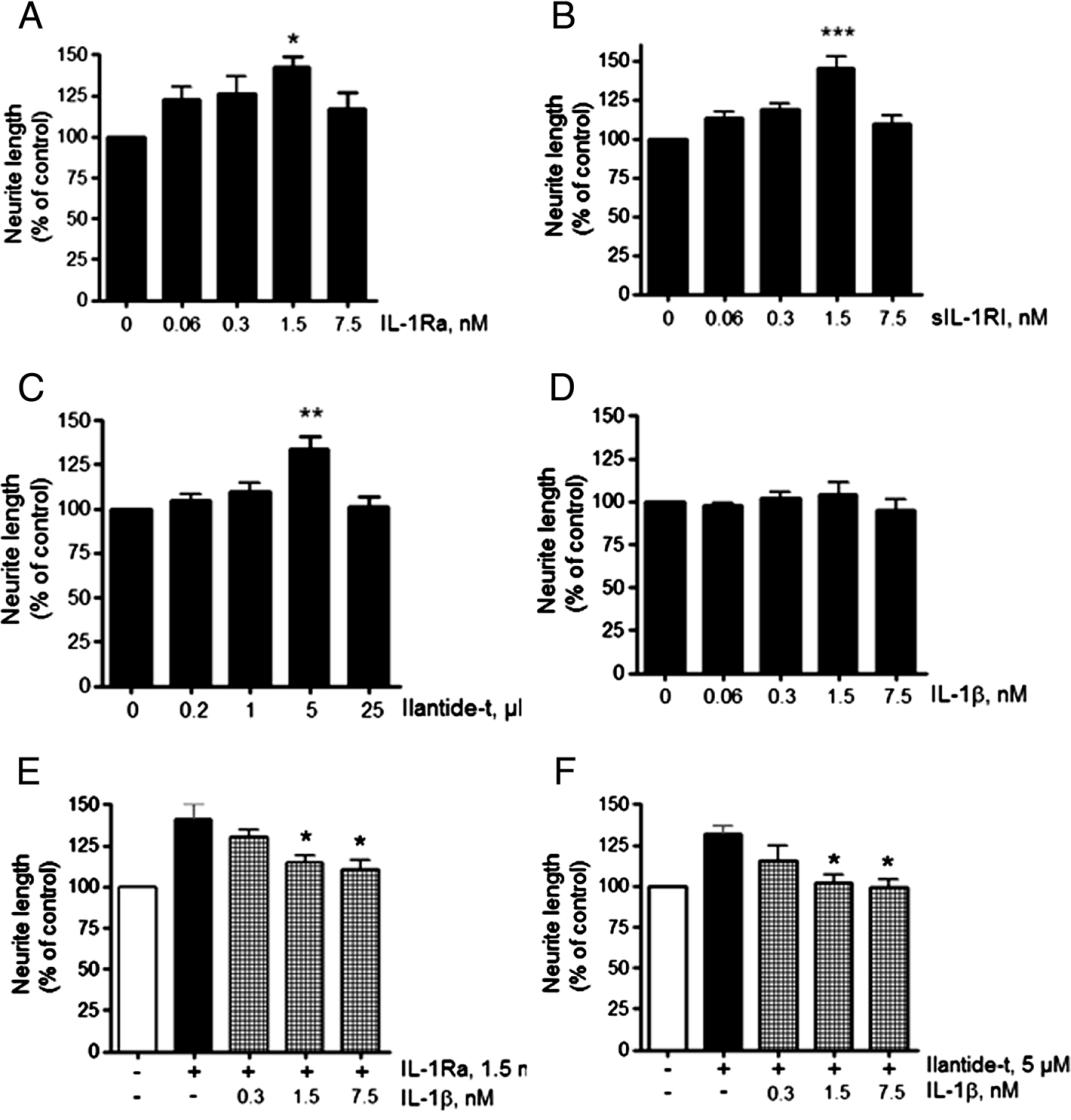
**Effect of Ilantide on neurite outgrowth from cerebellar granule neurons.** Cerebellar granule neuron cultures were treated with interleukin 1 receptor antagonist (IL-1Ra) **(A)**, soluble interleukin 1 receptor type I (sIL-1RI) **(B)**, Ilantide-t **(C)** or IL-1β **(D)** for 24 hours. The cultures were fixed and immunostained with rabbit anti-rat growth-associated protein 43 (anti-GAP-43) primary antibodies and then with secondary Alexa Fluor 488 goat anti-rabbit antibodies. **(E)** Effect of IL-1β on neurite outgrowth induced by IL-1Ra. **(F)** Effect of IL-1β on neurite outgrowth induced by Ilantide-t. The results from three to four independent experiments are expressed as percentage ± SEM with untreated controls set at 100%, corresponding to an average neurite length of 19.3 ± 4.1 μm. *P < 0.05 and **P < 0.01 compared with untreated controls (A to D) or cultures treated with either IL-1Ra or Ilantide alone (E and F) by one-way analysis of variance followed by the Newman-Keuls *post hoc* test).

IL-1RI is widely expressed in the central nervous system, including cerebellar, hippocampal and hypothalamic neurons [26,27]. The firing rate of anterior hypothalamic neurons is inhibited by IL-1β exposure [28]. To test the effects of Ilantide-t, IL-1β, IL-1Ra and sIL-1RI on neuronal differentiation, CGNs were grown on fibroblast cells in the absence or presence of these compounds at various concentrations. We found that Ilantide-t, IL-1Ra and sIL-1RI significantly induced neurite outgrowth from 7-day-old CGNs in a dose-dependent manner (Figures 6A to 6C), whereas IL-1β did not have neuritogenic effects (Figure 6D).

We subsequently tested whether IL-1β interferes with the neuritogenic effect of Ilantide and IL-1Ra. IL-1β at concentrations of 1.5 nM and 7.5 nM significantly inhibited the neuritogenic effect of both IL-1Ra and Ilantide-t (Figures 6E and 6F). Thus, the inhibition of IL-1R signaling resulted in the stimulation of neurite outgrowth.

### Ilantide protects rat pancreatic islets from interleukin 1β-induced apoptosis independently of nitric oxide

**Figure 7:**
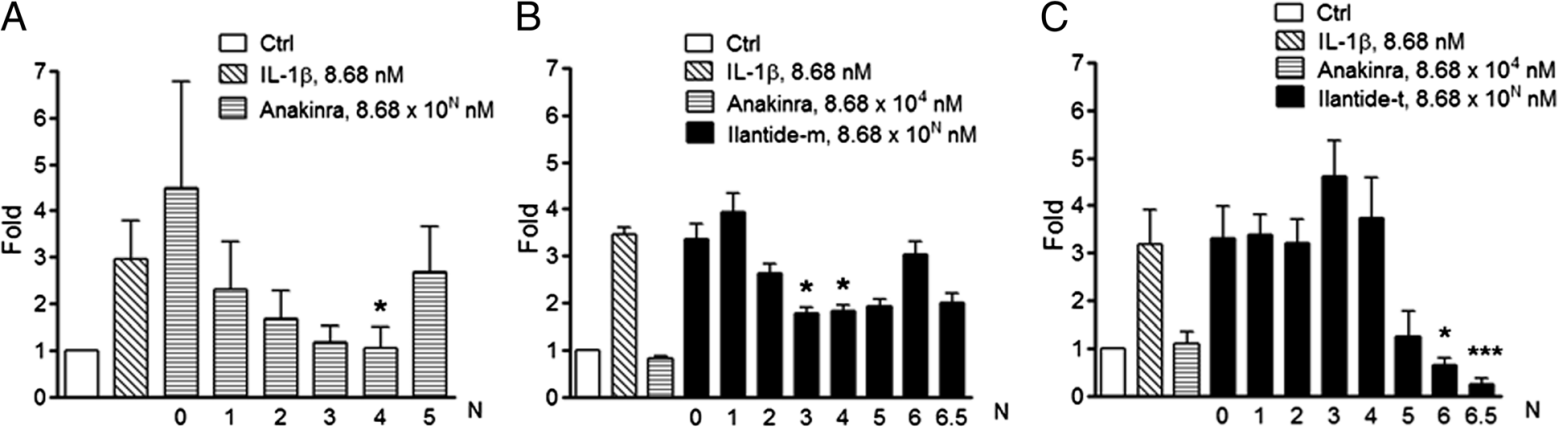
**Effect of Ilantide on interleukin 1β-induced rat islet cell apoptosis.** Rat pancreatic islets were preincubated for 30 minutes in the absence or presence of various concentrations of anakinra **(A)**, Ilantide-m **(B)** or Ilantide-t **(C)** and then exposed to 8.68 nM interleukin 1β (IL-1β) for 24 hours. Cytokine-induced apoptosis was determined by cell death detection enzyme-linked immunosorbent assay. Apoptosis rates are presented as fold induction compared with untreated control islets (white bars). The data from four independent experiments are presented as mean ± SEM. *P < 0.05 and **P < 0.01 compared with cultures treated with IL-1β alone (one-way analysis of variance followed by Dunnett’s *post hoc* test).

To investigate the effectiveness and potency of Ilantide in inhibiting IL-1-induced primary rat islet apoptosis, Ilantide was added at a concentration of 8.68 × 10^*n*^ nM as indicated prior to exposure to a known proapoptotic concentration of IL-1β of 8.68 nM (150 pg/ml) [29]. Consistent with previous findings [30], we observed that a maximally protective effect of Ana of 64% inhibition against IL-1β-induced apoptosis was obtained at a concentration of 8.68 × 10^4^ nM (Figure 7A), and this condition was used as a positive control in the experiments with Ilantide.

Ilantide-m maximally inhibited IL-1β-induced apoptosis by 49% at 8.68 × 10^3^ nM (Figure 7B). Ilantide-t was a more effective antagonist of IL-1β-induced apoptosis, with 93% inhibition, exceeding the antagonistic effect of Ana, although Ilantide-t had lower potency (8.68 × 10^6^ nM) (Figure 7C).

**Figure 8:**
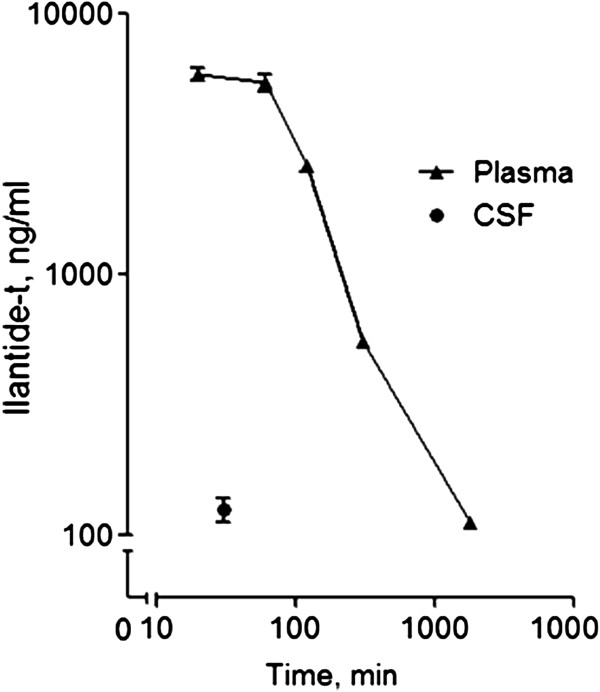
**Effect of Ilantide on interleukin 1β-induced nitric oxide production.** Anakinra **(A)** but none of the peptides, Ilantide-m **(B)** or Ilantide-t **(C)**, inhibited interleukin 1β (IL-1β)-induced nitric oxide production. Nitrites were measured in the supernatants using data derived from the experiments presented in Figure 7. The results of four independent experiments are shown. *P < 0.05 and **P < 0.01 compared with cultures treated with IL-1 alone (one-way analysis of variance followed by Dunnett’s *post hoc* test).

In addition to preventing IL-1β-induced β-cell apoptosis, Ana inhibits IL-1β-induced nitric oxide production [29]. Interestingly, none of the Ilantide peptides significantly inhibited IL-1β-induced nitric oxide production at the concentrations that reduced IL-1β-induced apoptosis (Figure 8).

### Ilantide crosses the blood–brain barrier

**Figure 9:**
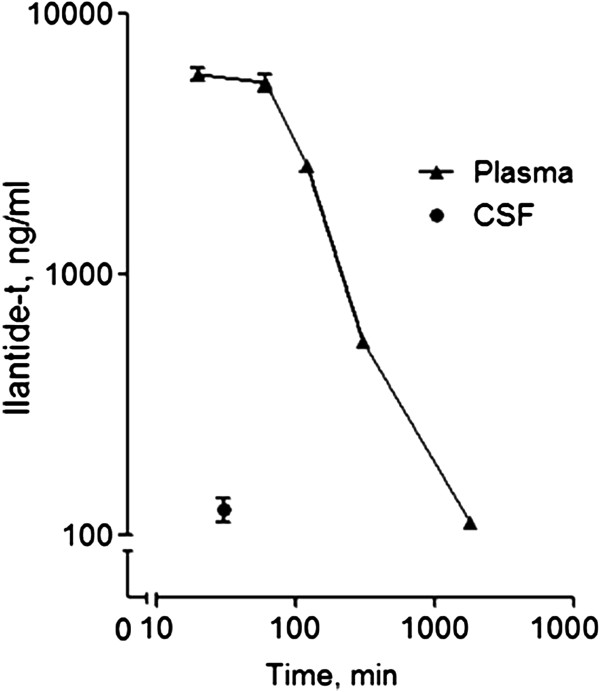
**Time course of Ilantide concentration in plasma and cerebrospinal fluid after a single subcutaneous injection of 10 mg/kg.** Peptide concentrations were measured by performing a competitive enzyme-linked immunosorbent assay. The data are expressed as mean ± SEM. CSF, cerebrospinal fluid.

The time course of the peptide concentrations in plasma samples collected from rats after a single subcutaneous injection at a 10 mg/kg dose is shown in Figure 9. Ilantide-t was detected in plasma 20 minutes after administration (5,816 ± 44 ng/ml) and remained detectable for up to 30 hours (111 ± 3.3 ng/ml). Twenty minutes after subcutaneous administration, the peptide concentration in plasma averaged approximately 6 μg/ml, and its half-life was calculated as approximately 2 hours. The peptide concentration in CSF was 125 ± 13 ng/ml 30 minutes after systemic administration. Thus, the ratio between the Ilantide concentration in plasma (measured at 20 minutes) and the Ilantide concentration in CSF (measured at 30 minutes) was 55.

### Ilantide reduces inflammation in collagen-induced arthritis model

**Figure 10:**
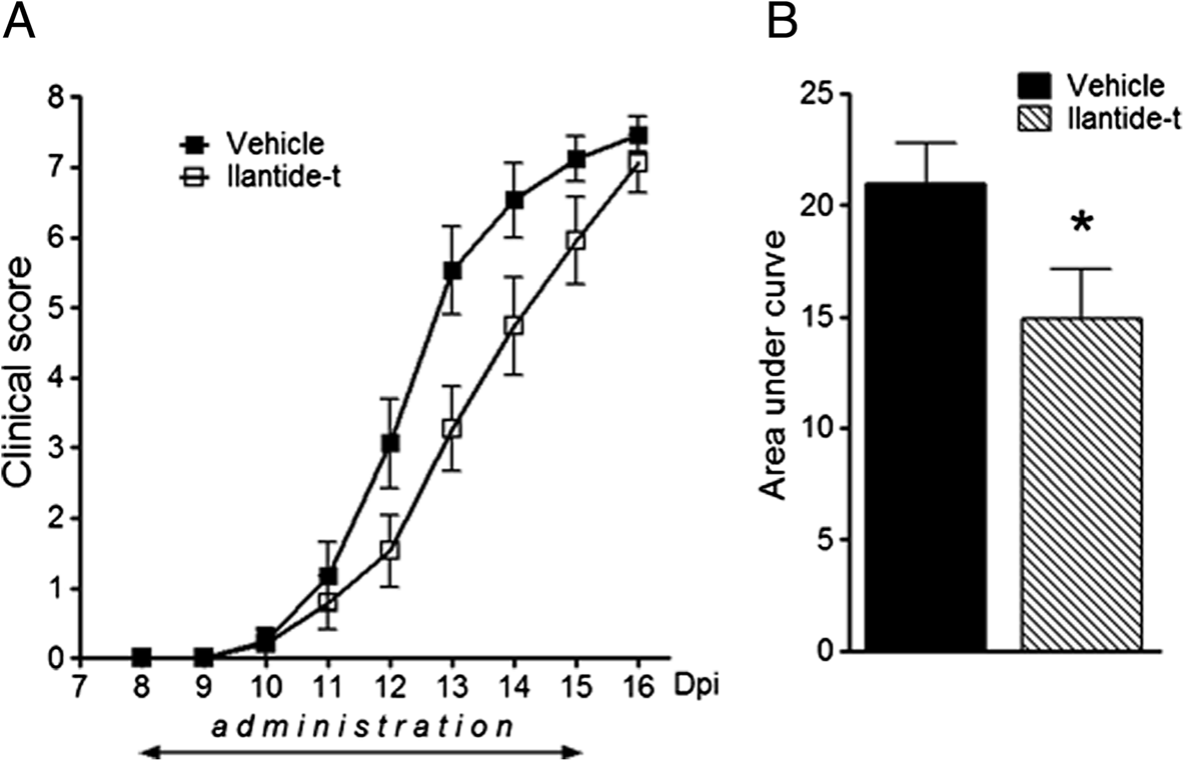
**Ilantide attenuates the severity of arthritis in animals with collagen-induced arthritis. (A)** Collagen-induced arthritis was induced by an intradermal injection of 250 μg of collagen II emulsified in complete Freund’s adjuvant (125 μg *M. tuberculosis*). On day postimmunization 8 (dpi 8), before the onset of clinical signs, all of the animals were randomly divided into two groups and subcutaneously dosed daily for 8 days (dpi 8 to 15) with 10 mg/kg Ilantide-t (*n* = 17) or 1 ml/kg phosphate-buffered saline vehicle (*n* = 16). **(B)** To evaluate cumulative clinical scores during the entire treatment period, the values are expressed as the area under the curve for each group during dpi 10 to 15 with the baseline set at dpi 8. The data are expressed as mean ± SEM. *P < 0.05 by unpaired *t*-test.

IL-1 is a major pathogenic cytokine in the development of rheumatoid arthritis [31]. The concentration of IL-1 in sera of rats with CIA is already elevated during the preclinical stage of the disease, and it reaches a maximal level during the first 4 to 5 days after disease onset [32]. Therefore, we used a treatment regimen that specifically covered this period. Figure 10 illustrates the dynamics of the clinical state (Figure 10A) and cumulative clinical scores (Figure 10B) in rats with CIA. Ilantide-t significantly attenuated the severity of CIA on dpi 10 to 16 compared with the vehicle-treated group (*t* = 2.1, *df* = 31; *P* < 0.05 by unpaired *t*-test).

### Ilantide strongly reduces lipopolysaccharide-induced behavioral changes

**Figure 11:**
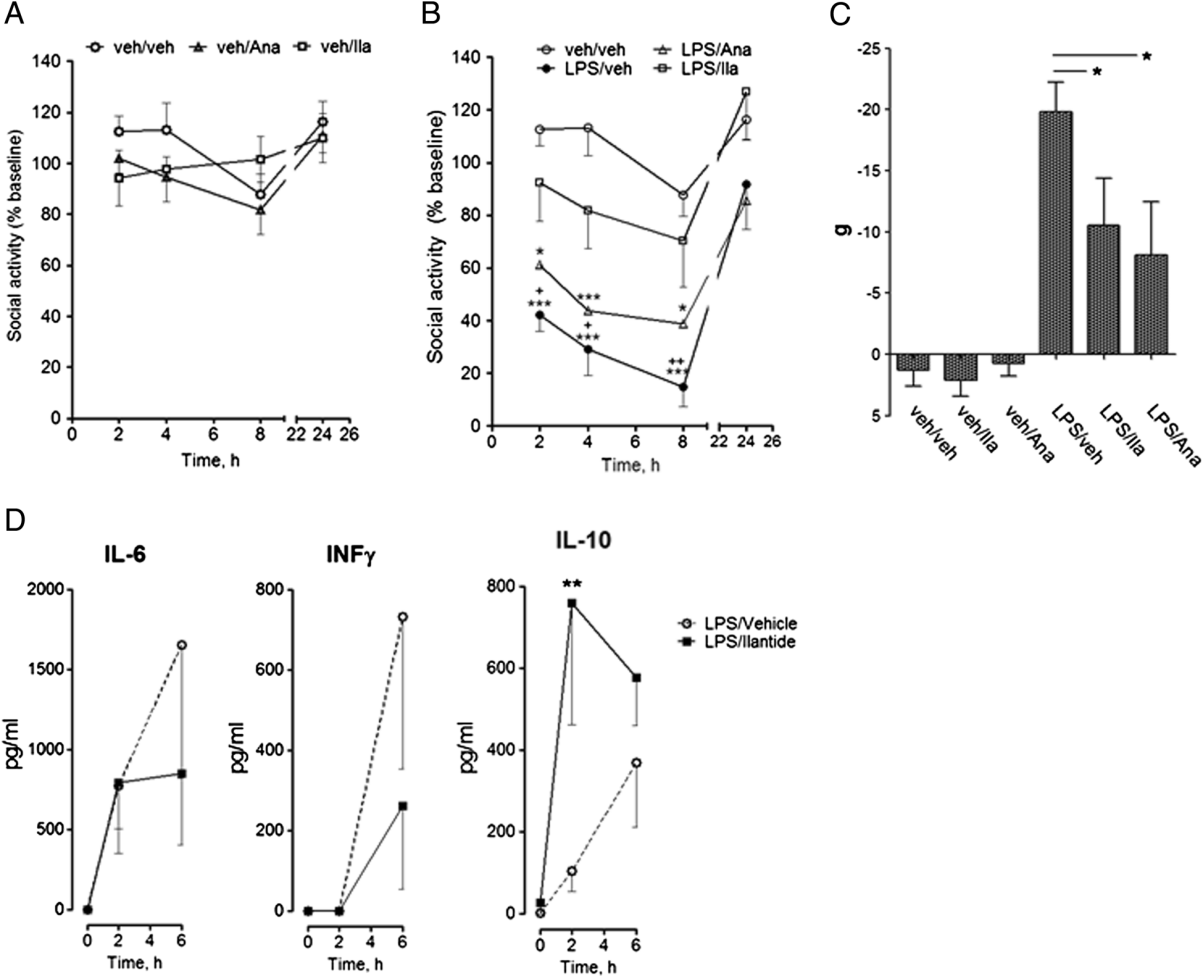
**Ilantide attenuates the decrease in social activity and increases plasma levels of interleukin 10 in lipopolysaccharide-induced neuroinflammation.** Ilantide-t (Ila; 10 mg/kg), anakinra (Ana; 100 mg/kg) or vehicle (Veh; 1 ml/kg) was subcutaneously injected simultaneously with an intraperitoneal injection of either lipopolysaccharide (LPS) (250 μg/kg) or vehicle (1 ml/kg). Baseline social activity was recorded for 4 minutes for all of the animals at 0 hours. The duration of social interaction at each time point is expressed as a percentage of baseline values and was averaged for each experimental group. The data are expressed as mean ± SEM. **(A)** Social activity of rats in control groups. **(B)** Social activity of rats in LPS-treated and veh/veh groups (SEM for LPS/Ana group not presented). *P < 0.05 and ***P < 0.001 compared with control (veh/veh). ^+^*P* < 0.05 and ^++^*P* < 0.01 compared with LPS/Ila group. **(C)** Weight gain is expressed as the difference from the body weight measured at 0 and 24 hours after LPS injection. *P < 0.05 (one-way analysis of variance (ANOVA) followed by Newman-Keuls *post hoc* test). **(D)** Plasma levels of interleukin 6 (IL-6), interferon γ and IL-10. **P < 0.01 (B and D), by two-way ANOVA followed by Bonferroni *post hoc* test.

Two-way ANOVA did not reveal significant differences between the veh/veh, veh/Ila and veh/Ana groups (*F*_2,72_ = 1.04, *P* = 0.37), indicating that Ilantide-t and Ana did not modify social behavior *per se* (Figure 11A). LPS significantly depressed social activity for 8 hours following compound administration (*F*_1,72_ = 54.59, veh/veh vs*.* LPS/veh; *P* < 0.0001). Ilantide-t counteracted the onset of sickness behavior and attenuated social depression compared with vehicle-treated animals (*F*_1,76_ = 13.92, LPS/veh vs*.* LPS/Ila; *P* = 0.001). No significant effect of Ana on sickness behavior was observed (Figure 11B).

One-way ANOVA showed that LPS decreased body weight 24 hours after LPS injection (*P* < 0.001, LPS/veh vs*.* Veh/Veh, Newman-Keuls *post hoc* test). Ilantide-t and Ana attenuated the anorexigenic effect of LPS (*P* < 0.05, LPS/veh vs*.* LPS/Ila and LPS/veh vs*.* LPS/Ana, Newman-Keuls *post hoc* test) (Figure 11C). The effect of Ilantide-t on LPS-induced sickness behavior was accompanied by an increase in IL-10 plasma levels 2 hours after LPS administration (*F*_1,42_ = 6.03, *P* < 0.01) (Figure 11D). There was a tendency toward reduction of levels of proinflammatory cytokines (IL-6 and IFN-γ) 6 hours following LPS injection, although the effect was not statistically significant (Figure 11D).

### Ilantide delays clinical signs of experimental autoimmune enchephalomyelitis

**Figure 12:**
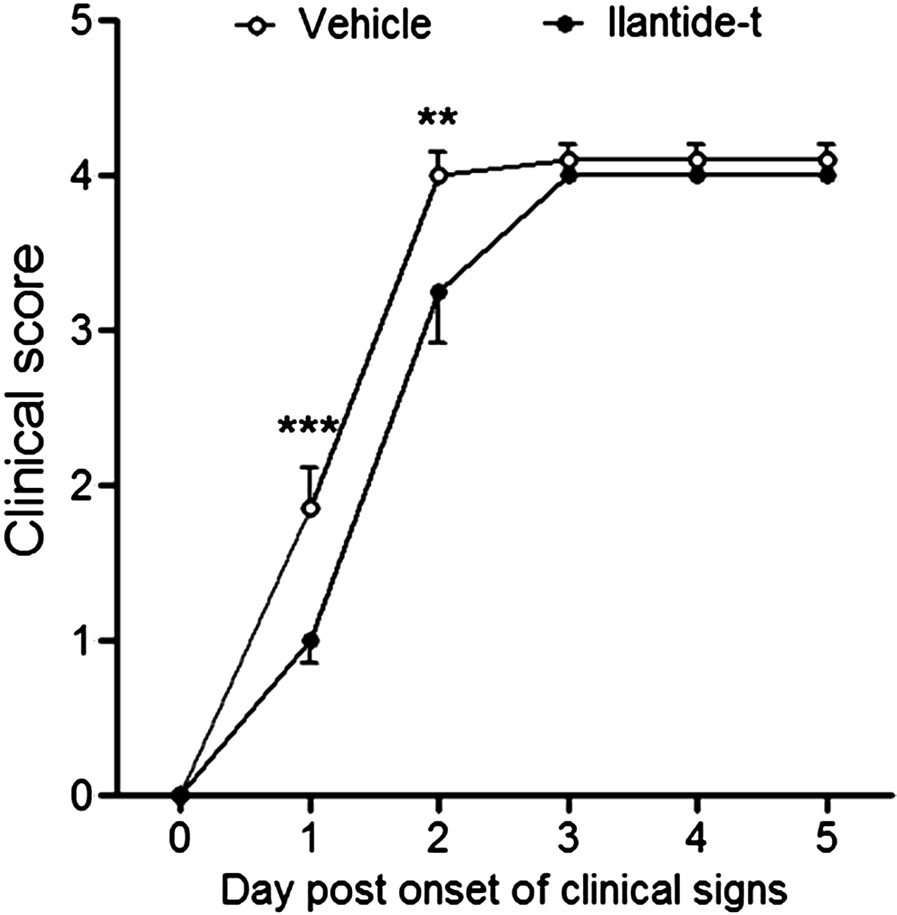
**Ilantide delays the progression of experimental autoimmune encephalomyelitis.** Experimental autoimmune encephalomyelitis (EAE) was induced in 30 animals, from among which 22 developed clinical signs of EAE (12 from the control group and 10 from the group treated with Ilantide-t). Clinical signs were evaluated daily. All of the data were aligned according to the onset of clinical signs and are presented as mean ± SEM. When the animals were killed, their clinical scores on the day they were killed were maintained at all subsequent time points. **P < 0.01 and ***P < 0.001 (two-way repeated-measures analysis of variance followed by the Bonferroni *post hoc* test).

To further investigate the effect of Ilantide on neuroinflammation, EAE (that is, an animal model of multiple sclerosis) was induced in rats by injecting MBP in CFA. Following the induction of EAE, the animals’ weight, clinical signs of EAE and survival were recorded. The first clinical signs of EAE appeared on dpi 10. From dpi 10 to 14, the animals were treated daily with either Ilantide or vehicle. Treatment with Ilantide-t had no significant effects on weight change or survival (data not shown). As shown in Figure 12, however, the clinical signs of EAE were attenuated by Ilantide-t. In Figure 12, the data from individual animals are aligned according to the first appearance of clinical signs. On the first and second days, the mean clinical signs were significantly lower in Ilantide-t-treated animals than in vehicle-treated animals. At later time points, however, no difference was found between the mean clinical signs in the two groups. These results show that the treatment of EAE with Ilantide attenuated, but did not prevent, the development of EAE.

### Ilantide reduces social memory deficits induced by amyloid-β

**Figure 13:**
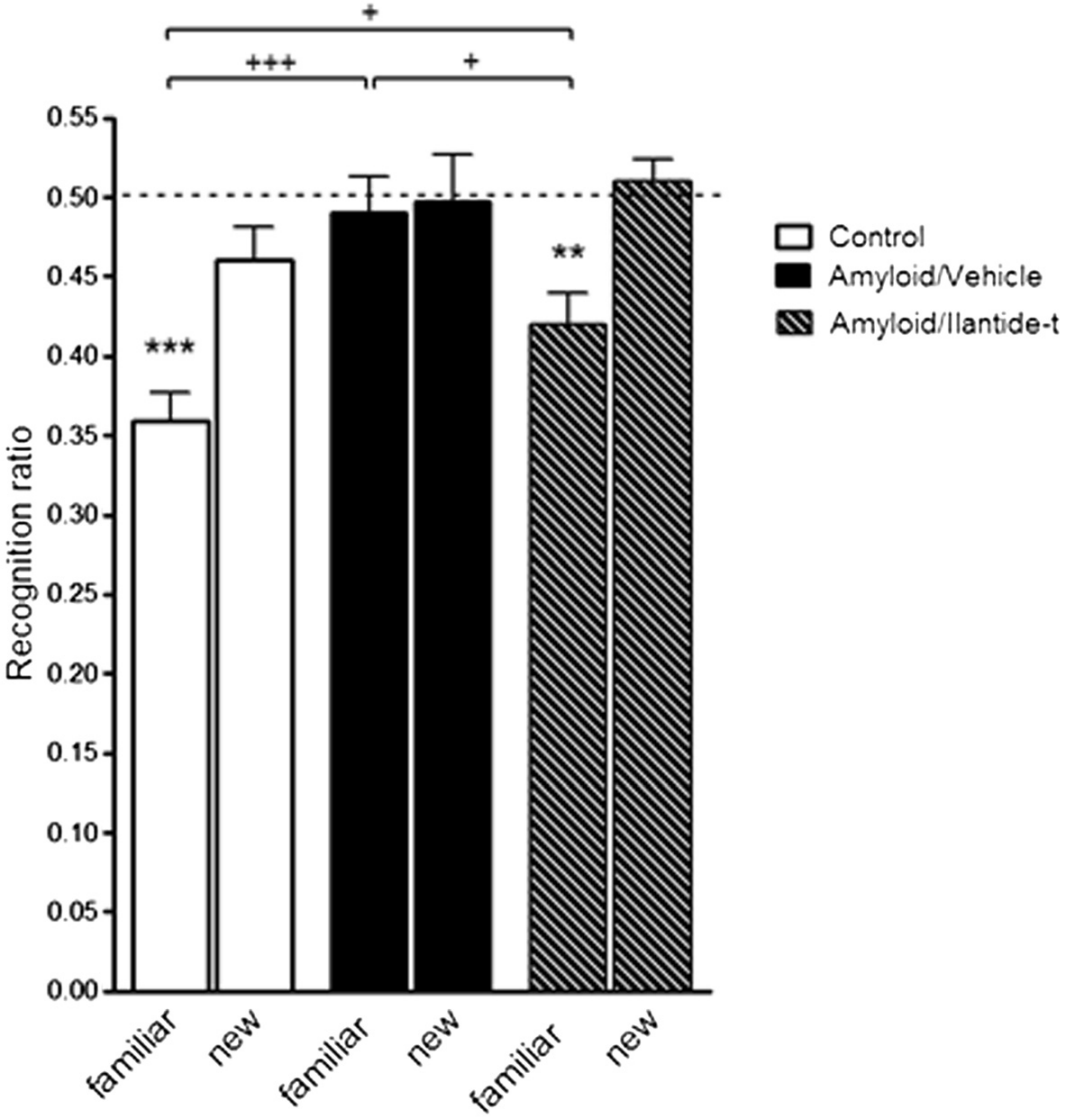
**Ilantide reduces social memory deficits induced by amyloid-β.** Ilantide-t (10 mg/kg; *n* = 12) or vehicle (saline, 1 ml/kg; *n* = 13) was subcutaneously injected on days postimmunization (dpi) 7, 9, 11, 13, 15, 17 and 19 after intracerebroventricular administration of aggregated Aβ_25–35_ (15 μg). The control group consisted of 11 rats. The social recognition test was performed on dpi 20. Social memory was estimated as the recognition ratio (RR). RR_familiar_ was calculated as T_2_/(T_1_ + T_2_). T_1_ and T_2_ are the times spent investigating the same juvenile animal during the first and second trials, respectively. RR_new_ was calculated as T_3_/(T_1_ + T_3_). T_1_ and T_3_ are the times spent investigating the different juvenile animals during the first and third trials, respectively. An RR value significantly less than the theoretical value of 0.5 obtained by one-sample *t*-test was taken as an indication of the presence of social memory. **P < 0.01 and ***P < 0.001 by one-sample *t*-test compared with theoretical value of 0.5. ^+^*P* < 0.05 and ^+++^*P* < 0.001 by one-way analysis of variance followed by the Newman-Keuls *post hoc* test.

Aβ is known to induce deficits in social memory. Therefore, we tested whether Ilantide can rescue Aβ-induced social memory impairment. The rats were administered Aβ_25–35_, followed by administration with either Ilantide-t or vehicle. No significant intergroup difference in social activity was observed during the first trial, suggesting that Aβ and Ilantide-t did not influence investigatory activity *per se* (*F*_2,32_ = 0.94; *P* = 0.4 by one-way ANOVA) (data not shown). Social memory was measured as a decrease in the time that an adult rat spent investigating the same (that is, familiar) juvenile during the second trial compared with the first trial. The social recognition RR_familiar_ value was <0.5 in the untreated group (*t* = 7.67, *df* = 10; *P* < 0.0001 by one-sample *t*-test) (Figure 13). Animals in the Aβ/veh group exhibited a significant deficit in social memory. The rats in this group failed to recognize the juvenile during the second trial. RR_familiar_ did not differ from 0.5 (*t* = 0.44, *df* = 11; *P* = 0.67 by one-sample *t*-test) and significantly differed from the RR_familiar_ in the control group (*P* < 0.001 by one-way ANOVA followed by the Newman-Keuls *post hoc* test). Treatment with Ilantide-t counteracted the development of cognitive impairment. The RR_familiar_ in this group was significantly lower than the theoretical value of 0.5 (*t* = 4.12, *df* = 11; *P* = 0.002 by one-sample *t*-test) and the value in the Aβ/veh group (*P* < 0.05 by one-way ANOVA followed by the Newman-Keuls *post hoc* test). The Aβ/Ilantide-treated rats were still able to recognize a new, unfamiliar juvenile during the third trial (that is, the RR_new_ value did not differ from 0.5) (*t* = 0.7, *df* = 11; *P* = 0.5 by one-sample *t*-test), indicating that Ilantide ameliorated the social memory deficit in Aβ-treated animals.

## Discussion

A critical role for IL-1 in the pathogenesis of acute and chronic inflammation has been well-documented [33-35]. IL-1RI blockade is one of the current strategies used in the treatment of IL-1-induced conditions [1], and an IL-RI receptor antagonist, Ana, has already been approved for the treatment of some of these conditions. Despite the high efficacy of Ana, however, this drug has some disadvantages, such as a short-lasting therapeutic effect in 20% of patients with rheumatoid arthritis [36], local injection reactions (for example, erythema, ecchymosis, inflammation and pain [37]) and in some cases depression [38]. Furthermore, the absence of an effect of peripheral administration of Ana on neuroinflammation observed in the present study should be added to the list of Ana’s disadvantages, although further testing with other Ana doses is needed to substantiate this finding.

The aim of the present study was to develop a synthetic low-molecular-weight antagonist of IL-1RI that is effective in the treatment of not only peripheral inflammation but also neuroinflammation. We found that a peptide termed *Ilantide*, derived from the *N* terminus of human IL-1Ra, mimics important characteristics of IL-1Ra. Thus, Ilantide binds to IL-1RI with an affinity comparable to that of IL-1Ra and with higher affinity than IL-1β. Importantly, Ilantide inhibits the binding of IL-1β to IL-1RI. Signaling from the IL-1/IL-1RI/Il-1RAcP complex is known to activate the NF-κB pathway [20]. We found that Ilantide inhibited NF-κB activation. We also found that Ilantide, similarly to IL-1Ra, inhibited the secretion of TNF-α by macrophages, thus antagonizing an important mechanism of the proinflammatory action of IL-1.

IL-1 induces apoptosis in insulin-producing pancreatic β-cells, and the administration of Ana in patients with type 2 diabetes mellitus improves β-cell function [39]. We investigated the protective potency of Ilantide against the IL-1-induced apoptosis of pancreatic islets and compared it to the potency of Ana. Ilantide-t in high molar excess reduced IL-1-induced apoptosis by 93%, which was 1.5× the maximal effect of Ana. Unlike Ana, however, the effect of Ilantide was not associated with inhibition of IL-1-induced nitric oxide production, which is consistent with the less effective inhibition of NF-κB activation compared with IL-1Ra (Figures 3A and 3C). The molecular mechanism that underlies this discrepancy is unclear, but it may be related to the lower steric hindrance that Ilantide exerts on the IL-1/IL-1RT1/IL-1RAcP interaction complex compared with IL-1Ra, thus allowing the escape of certain parts of IL-1 signaling. IL-1Ra is known to interact with all three IL-1RI receptor domains, whereas the Ilantide sequence motif includes only two residues from the binding site that is involved in the interaction with the Ig3 domain (see Figure 1B). Signaling via IL-1RI occurs only if the ligand-bound receptor associates with the IL-1RAcP protein. Two loops in IL-1Ra (β4–β5 and β11–β12) are determinants of the antagonism of IL-1Ra that prohibit ligand-bound IL-1RI to recruit Il-1RAcP [40]. The Ilantide motif is far from these two loops, and the actual mechanism of the Ilantide-induced inhibition of IL-1 signaling might be somewhat different from that of Ana. From the pharmaceutical perspective, the chemically produced Ilantide peptide has a number of advantages over Ana (which belong to recombinant biologics), such as better penetration through the BBB, better stability and less batch-to-batch variations.

Rheumatoid arthritis is triggered and maintained by cascades of inflammatory mediators. The current treatments for RA include neutralizing antibodies and antagonists of mediators of inflammation, particularly IL-1 and TNF-α. We observed a positive effect of Ilantide in the CIA model that resembled several aspects of rheumatoid arthritis [41] when treatment began in the preclinical stage that corresponds to the first elevation of IL-1 levels in the blood. This result suggests that the blockade of IL-1 signaling with Ilantide may be an effective treatment for rheumatoid arthritis patients.

IL-1 receptors are well-known to be expressed in the brain [42,43], and IL-1 is a key mediator of neuroinflammation. IL-1β is synthesized and released by microglia and astrocytes and regulates IL-1RI expression in neurons and astrocytes [44]. The neuron-specific effects of IL-1β were recently shown to be mediated by a novel isoform of IL-1RAcP—IL-1RAcPb, which results in the activation of p38MAPK, but not NF-κB, in hippocampal neurons [45]. However, the role of neuronal p38MAPK signaling is poorly understood [46]. We found that both Ilantide and recombinant IL-1Ra induced neuritogenesis and promoted the survival of primary neurons. The neuronal cultures used in our experiments did not contain microglial cells [47] and included less than 5% astrocytes, which could be a source of IL-1 that affects the ability of neurons to differentiate and survive. Ilantide and IL-1Ra likely inhibited the effect of traces of IL-1 on IL-1RI, thereby triggering neuritogenesis and survival mechanisms.

Intraperitoneal injections of LPS have been shown to induce IL-1 production in peripheral activated monocytes and macrophages, as well as in the brain (that is, the hypothalamus and hippocampus) [48]. IL-1 overproduction induces anorexia and depression, causing body weight loss and a reduction of social activity in rodents. One injection of Ilantide significantly ameliorated these effects of LPS, a potent inducer of IL-1 production. This peptide effect was accompanied by an increase in the plasma levels of the anti-inflammatory cytokine IL-10 in LPS-treated animals. We have shown in the present study that Ilantide crossed the BBB, which explains the protective effects of Ilantide on neuroinflammation. The ratio between Ilantide concentration in plasma and its concentration in CSF was 55, which is in good agreement with our previous results obtained for other peptides, the peptide derived from fibroblast growth factor receptor (plasma/CSF ratio = 10) [49] and the peptide derived from the NCAM (plasma/CSF ratio = 40) [50]. Reasonable BBB penetration of the peptide can also explain the cerebroprotective effect of Ilantide in the model of AD. IL-1 contributes to neuroinflammation during the progression of AD. Intracerebroventricular administration of aggregated Aβ_25–35_ induces neuroinflammation, which is manifested by the activation of microglia and astrocytes, neuronal damage and cognitive impairments [51] and overproduction of IL-1 in activated microglia [52]. Our *in vitro* data show that Ilantide promoted neural survival and neuritogenesis, which is an essential component of neuroplasticity and can explain the mnemotropic effect of Ilantide in the AD model.

EAE is often used as a model of multiple sclerosis in humans. The activation of endogenous IL-1 is involved in the progression of EAE, and IL-1Ra can reduce the clinical signs of the disease [53,54]. Comparisons of the beneficial effects of IL-1Ra and Ilantide showed that a more profound effect of IL-1Ra was achieved by a 26× higher total dose per kilogram compared with Ilantide in the present study [54]. Others have shown that the same dose [55] or a lower dose [56] of IL-1Ra has no significant effect on clinical scores.

In conclusion, we have identified a novel 10–amino acid peptide antagonist of IL-1, Ilantide, that binds to IL-1RI and inhibits the IL-1-induced activation of NF-κB and secretion of TNF-α by macrophages. The peptide protected pancreatic islets from IL-1β-induced apoptosis and reduced inflammation in CIA. The Ilantide peptide penetrated the BBB, diminished the deficit in social activity and memory in LPS- and amyloid-induced neuroinflammation and delayed the development of EAE in rats. We propose that this novel IL-1 antagonist can be used as an alternative to other IL-1 blockers to inhibit a variety of inflammatory conditions.

## Abbreviations

AD: Alzheimer’s disease; BBB: Blood–brain barrier; CIA: Collagen-induced arthritis; CSF: Cerebrospinal fluid; EAE: Experimental autoimmune encephalomyelitis; IFN: Interferon; IL: Interleukin; IL-1RAcP: Interleukin 1 receptor accessory protein; IL-1RI: Interleukin 1 receptor I; LPS: Lipopolysaccharide; MAPK: Mitogen-activated protein kinase; NF-κB: Nuclear factor κB; RA: Rheumatoid arthritis; SEAP: Secreted embryonic alkaline phosphatase; STAT: Signal transducer and activator of transcription; TNF: Tumor necrosis factor.

## Competing interests

EB and VB are shareholders in Phlogo ApS (Denmark), which owns a patent on the Ilantide peptide. This does not alter the authors’ adherence to all *Journal of Neuroinflammation* policies on sharing data and materials.

## Authors’ contributions

BK participated in the design of *in vivo* studies; carried out *in vivo* experiments on LPS, CIA and AD; and drafted the manuscript. SL carried out the binding and TNF secretion studies. IK carried out the pharmacokinetics study. OD and SP participated in study design and carried out the EAE and ELISA studies. PSW participated in study design and the coordination of EAE study and helped to draft the manuscript. LKK carried out the pancreatic islets study and helped to draft the manuscript. MSD, ML and DPC participated in the pancreatic islets study and statistical analysis. TMP designed and coordinated the pancreatic islets study and participated in drafting the manuscript. EB participated in designing the *in vitro* and *in vivo* studies and helped to draft the manuscript. VB designed and coordinated the whole project and, together with BK, drafted the manuscript. All authors read and approved the final manuscript.
